# Chymase-producing cells of the innate immune system are required for decidual vascular remodeling and fetal growth

**DOI:** 10.1038/srep45106

**Published:** 2017-03-22

**Authors:** Nicole Meyer, Katja Woidacki, Martin Knöfler, Gudrun Meinhardt, Désirée Nowak, Philipp Velicky, Jürgen Pollheimer, Ana C. Zenclussen

**Affiliations:** 1 Experimental Obstetrics and Gynecology, Medical Faculty, Otto-von-Guericke University, Magdeburg, Germany; 2 Dept. of Obstetrics and Fetal-Maternal Medicine, Medical University of Vienna, Vienna, Austria

## Abstract

Intrauterine growth restriction (IUGR) is caused by insufficient remodeling of spiral arteries (SAs). The mechanism underlying the relevance of natural killer cells (NKs) and mast cells (MCs) for SA remodeling and its effects on pregnancy outcome are not well understood. We show that NK depletion arrested SA remodeling without affecting pregnancy. MC depletion resulted in abnormally remodeled SAs and IUGR. Combined absence of NKs and MCs substantially affected SA remodeling and impaired fetal growth. We found that α-chymase mast cell protease (Mcpt) 5 mediates apoptosis of uterine smooth muscle cells, a key feature of SA remodeling. Additionally, we report a previously unknown source for Mcpt5: uterine (u) NKs. Mice with selective deletion of Mcpt5^+^ cells had un-remodeled SAs and growth-restricted progeny. The human α-chymase CMA1, phylogenetic homolog of Mcpt5, stimulated the *ex vivo* migration of human trophoblasts, a pre-requisite for SA remodeling. Our results show that chymases secreted by uMCs and uNKs are pivotal to the vascular changes required to support pregnancy. Understanding the mechanisms underlying pregnancy-induced vascular changes is essential for developing therapeutic options against pregnancy complications associated with poor vascular remodeling.

Fetal development is a complex process that requires the coordinated activity of the cardiovascular system to proceed efficiently. Continuous remodeling of spiral arteries (SA), the term branches of the uterine artery, from thick- to thin-walled arteries during pregnancy is essential to ensure a consistent blood supply to the fetus and its proper development. This remodeling process enables a 10-fold increase in fetal blood supply^1^ and is driven by smooth muscle cell (SMC) apoptosis^2^. Inadequate SA remodeling is associated with late miscarriage, preeclampsia or fetal growth restriction^2^,^3^,^4^. Small size at birth following suboptimal SA remodeling may lead to serious complications in adulthood, such as the risk of developing cardiovascular or metabolic diseases^5^. Uterine natural killer cells (uNKs), the most abundant immune cells at the feto-maternal interface, were showed to be associated with SA remodeling^6^. uNKs proliferate at the beginning of pregnancy, reach their highest numbers at midgestation and then decline^7^,^8^. They reportedly regulate trophoblast invasion in the endometrium and are involved in placental development^9^,^10^,^11^. However, their absence does not dramatically impair pregnancy outcomes^7^. This finding supports the existence of redundant mechanisms that ensure the fulfillment of important processes, such as SA shaping, to guarantee blood supply from the mother to the fetus. We recently reported that uterine mast cells (uMCs) are involved in SA remodeling^12^ after observing that MC-deficient C57BL/6J-Kit^W-sh/W-sh^ (W-sh) mice showed impaired SA remodeling that could be restored after reconstitution of the mice with bone marrow derived mast cells (BMMCs)^12^,^13^. The uterine MC population consisting of connective tissue type MCs (CTMCs) and mucosal type MCs (MMCs), accumulates in the uterus during sexual receptivity and increases in number after fertilization^12^. As both uMCs and uNKs appear to be of importance for an efficient SA remodeling process; we embarked on a study that aimed to investigate the consequences of the combined absence of uNKs and uMCs for SA remodeling and fetal development. We further sought to unravel the potential mediators of their action and to understand whether this mechanism is relevant for human pregnancies.

Proteases secreted by MCs can be divided into carboxypeptidase A3 (Cpa3), tryptases and chymases; the latter are serine proteases that can be further divided into α- and β-chymases^14^. Whereas CTMCs predominantly expresses the murine α-chymase mast cell protease (Mcpt) 5, the β-chymases Mcpt-1, −2 and −4 are expressed by MMCs. The only human chymase is the α-chymase CMA1^15^,^16^, the phylogenetic homolog of mouse Mcpt5^17^. Chymases are able to regulate the blood pressure^18^ and activate matrix-metalloprotease (MMP) precursors MMP9 and MMP2^19^,^20^,^21^. Additionally chymases degrade extracellular matrices^22^,^23^, inhibit the proliferation of vascular SMCs^24^ and induce apoptosis in vascular SMCs^22^,^25^,^26^. These functions suggest a possible role of chymase during SA remodeling. To analyze the possible role of chymase, as putative mediator of MCs and eventually NKs in SA remodeling was the second aim of this study.

We generated a mouse model that lacks both NKs and MCs to examine the requirement of these cells for murine SA remodeling and pregnancy outcomes. Additionally we analyzed a possible role of chymase in SA remodeling-associated processes. We found that the combined absence of uNKs and uMCs substantially impaired SA remodeling and had detrimental consequences for fetal growth in contrast with no effect after NK depletion and a moderate effect in the absence of MCs. Further, we found that Mcpt5-expressing cells induce apoptosis of uterine SMCs (uSMCs) in the mouse system. CMA1 (human chymase)-expressing cells and human recombinant CMA1 increase the migration of invasive trophoblasts (EVTs). Both SMC apoptosis and increased EVT migration are reportedly important for SA remodeling. Additionally, we detected uNKs as a previously unreported source for the chymase Mcpt5.

## Results

### Depletion of NKs and generation of MC- and NK/MC-double-deficient mice

We generated NK-deficient mice using antibody-depletion strategies. First, we employed an anti-asialo GM1 antibody that resulted in a statistically significant (*P* < 0.05, *P* < 0.01) reduction in CD122^+^CD3^−^NK1.1^+^ pNKs (Fig. S1A–C) in the spleen, blood, and inguinal lymph nodes (LN). However, comparable numbers of DBA lectin-stained uNKs in implantations of anti-asialo GM1- and serum-treated control mice indicated that the antibody was not able to deplete DBA^+^ uNKs when it injected i.p. at a concentration of 0.29 mg/mouse every second day from gd0 to gd8 (Fig. S1D,i,ii,iv,v). This antibody was therefore not a choice of model for our project.

Treatment with anti-CD122 achieved a comparable efficient depletion in the number of CD122^+^CD3^−^NK1.1^+^ NKs in the spleen, blood, and inguinal LN at gd10 as anti-asialo GM1 (Fig. S1A–C). The complete absence of uNKs confirmed the effectiveness of the antibody in depleting DBA^+^ uNKs (Fig. S1D,iii,vi). This result validated the use of this antibody in our study. Thus, as anti-asialo GM1 treatment depleted peripheral (p) NKs but not uNKs, we used anti-CD122 to deplete both NK populations (Fig. S1). We cannot however not exclude that other ILCs, e.g. ILC1, were not affected by the treatment as they also express CD122.

**Figure 1:**
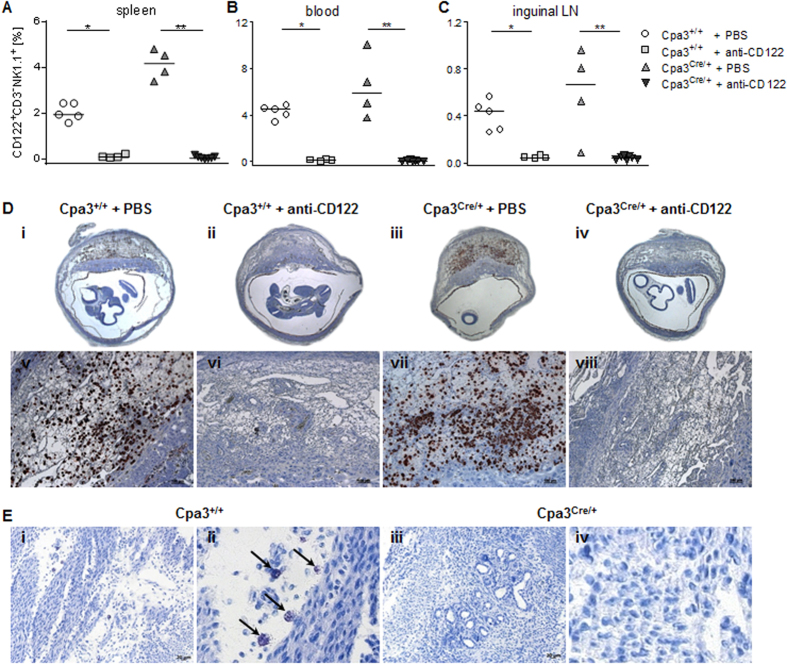
Anti-CD122 treatment depleted peripheral and uterine NKs in control Cpa3^+/+^ and MC-deficient Cpa3^Cre/+^ mice. The percentage of CD122^+^CD3^−^NK1.1^+^ pNKs in the spleen **(A)**, blood **(B)** and inguinal LN **(C)** at gd10 was analyzed by flow cytometry in samples from PBS- or anti-CD122-treated Cpa3^+/+^ and Cpa3^Cre/+^ mice. Results are presented as medians; shown are individual values for each mouse. Statistical analysis was performed with the Mann-Whitney *U* test (*P < 0.05, **P < 0.001). **(D)** Representative DBA lectin-stained cross sections of implantations from PBS- or anti-CD122-treated Cpa3^+/+^ and Cpa3^Cre/+^ mice at gd10 are depicted (upper panels, i–iv, 10×). Magnifications of the upper panels show uNKs, which are DBA lectin-positive, brown-stained cells within the *decidua basalis* (lower panels, v-viii, scale bars = 100 μm). **(E)** Representative toluidine blue-stained cross sections of the uterus from non-pregnant Cpa3^+/+^ (i) and Cpa3^Cre/+^ (iii) mice (scale bars = 20 μm) with close-ups (ii,iv) that show uMCs (arrows) in Cpa3^+/+^ mice but not in Cpa3^Cre/+^ mice. DBA, *Dolichos biflorus* agglutinin; gd, gestation day; LN, lymph nodes; p, peripheral; u, uterine.

Cpa3^Cre/+^ mice^27^ in which the Cre-recombinase inserted into the *Cpa3* locus is cytotoxic to Cpa3-producing cells were included as MC deficient mice as they lack CTMCs and MMCs^27^; Cpa3^+/+^ mice were included as MC sufficient controls. NK/MC-double-deficient mice were generated by treating MC-deficient Cpa3^Cre/+^ females with anti-CD122 (Fig. 1). PBS or anti-CD122-treated Cpa3^+/+^ mice and PBS-treated Cpa3^Cre/+^ mice served as further controls. Depletion of pNKs and uNKs with anti-CD122 in the Cpa3^Cre/+^ and Cpa3^+/+^ mice was confirmed 10 days after application (Fig. 1A–D). Anti-CD122-treated mice had no CD122^+^CD3^−^NK1.1^+^ pNKs in the spleen, blood and inguinal LN at gestation day (gd) 10 (Fig. 1A–C). Not a single uNK was observed in DBA lectin-stained sections of implantations from the anti-CD122-treated mice compared with the PBS-treated controls (Fig. 1D) in both MC-sufficient and MC-deficient mice. Immunohistochemistry of the uterus samples confirmed that uMCs were present in the Cpa3^+/+^ mice but absent in the Cpa3^Cre/+^ mice (Fig. 1E). Females of all groups had comparable implantation numbers (Fig. S2).

### Impaired spiral artery remodeling in NK-depleted and MC- and NK/MC-deficient mice

**Figure 2:**
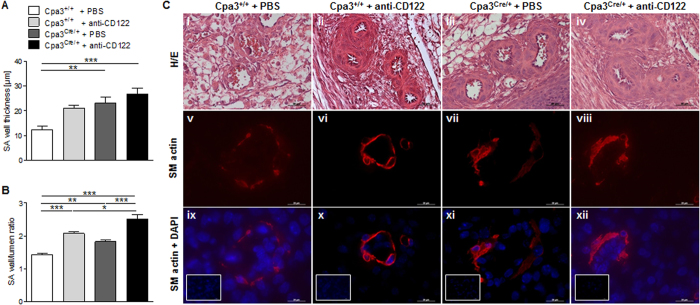
Impaired spiral artery remodeling in uteri of NK-depleted, MC- and NK/MC-deficient mice. SA wall and lumen diameters from 3 to 10 SAs per mouse in the Cpa3^+/+^  + PBS (*n* = 9), Cpa3^+/+^  + anti-CD122 (*n* = 4), Cpa3^Cre/+^  + PBS (*n* = 8) and Cpa3^Cre/+^  + anti-CD122 (*n* = 6) groups were measured, and the wall thickness **(A)** and wall-to-lumen ratios **(B)** were calculated. Results are presented as means ± SEM. Data was analyzed by using One-way ANOVA followed by Bonferroni post-test (*P < 0.05, **P < 0.01, ***P < 0.001). **(C)** Representative images of SAs in gd10 implantation samples, H/E-stained (i–iv, scale bars = 50 μm) or immunofluorescent-stained for SM actin (v–viii), SM actin in combination with DAPI (ix–xii, both scale bars = 20 μm) or DAPI (rectangles). gd, gestation day; SA, spiral artery; SM, smooth muscle.

To analyze pregnancy outcome in in NK-depleted and MC- and NK/MC-deficient mice, SA remodeling status was assessed. The quantitative measurement of SAs revealed a statistically significant increased wall thickness in Cpa3^Cre/+^ MC-deficient mice (*P* < 0.01) and NK/MC-deficient mice (*P* < 0.001) (Fig. 2A) and statistically significant increased wall-to-lumen ratio in NK-depleted mice (*P* < 0.001), MC-deficient mice (*P* < 0.01) and Cpa3^Cre/+^ MC-deficient mice (*P* < 0.001) (Fig. 2B). The most dramatic effects on SA remodeling were observed in NK/MC-deficient mice lacking both uNKs and uMCs. They had a very narrow SA lumen and thick arterial walls (Fig. 2C,iv). SM actin, which is expressed by vascular SMCs and used as an indicator of SMC layer thickness, was strongly expressed in the SA walls of the NK-depleted mice (Fig. 2C,vi,x), MC-deficient mice (Fig. 2C,vii,xi) and NK/MC-deficient mice (Fig. 2C,viii,xii) compared with controls (Fig. 2C,v,ix), confirming the poor vascular remodeling in mice lacking uNKs or uMCs.

### Maternal blood pressure in NK-depleted and MC- and NK/MC-deficient mice

**Figure 3:**
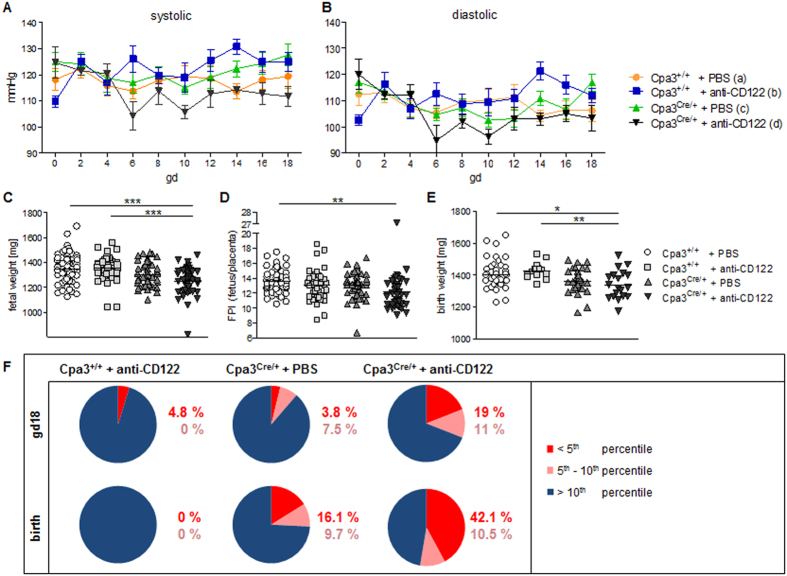
Gestational blood pressure and intrauterine growth in NK-depleted and MC- and NK/MC-deficient mice. Systolic **(A)** and diastolic **(B)** blood pressure of pregnant Cpa3^+/+^ mice treated with PBS (*n* = 9) or anti-CD122 (*n* = 5) and pregnant Cpa3^Cre/+^ mice treated with PBS (*n* = 8) or anti-CD122 (*n* = 4). Data were recorded every other day from gd0 to gd18. Results are presented as means ± SEM. Fetal weights **(C)** and the FPI **(D)** from the progeny of PBS- (*n* = 64) or anti-CD122-treated (*n* = 42) Cpa3^+/+^ females and PBS- (*n* = 53) or anti-CD122-treated (*n* = 42) Cpa3^Cre/+^ females at gd18. **(E)** Birth weights of the pups from PBS- (*n* = 49) or anti-CD122-treated (*n* = 15) Cpa3^+/+^ females and PBS- (*n* = 31) or anti-CD122-treated (*n* = 19) Cpa3^Cre/+^ females. In (**C**–**E)**, results are presented as individual values for each fetus or pup and means. Statistical differences were determined using One-way ANOVA followed by Bonferroni post-test (*P < 0.05, **P < 0.01 and ***P < 0.001). **(F)** Percentage of fetuses (upper panel, from **C**) or pups (lower panel, from **E**) from anti-CD122-treated Cpa3^+/+^ and PBS- or anti-CD122-treated Cpa3^Cre/+^ females whose weight was below the 5^th^ percentile (red); between the 5^th^ and the 10^th^ percentile (pink); or above the 10^th^ percentile (blue). FPI, feto-placental index; gd, gestation day.

Poor SA remodeling and maternal hypertension are often observed in pre-eclamptic patients^3^. We therefore asked whether the narrower SA lumen in the NK-depleted and MC- and NK/MC-deficient mice lead to maternal hypertension. To do this, we monitored systolic (Fig. 3A) and diastolic (Fig. 3B) maternal blood pressure from all groups throughout pregnancy using a tail-cuff system. However, none of the groups developed hypertension (>140 mmHg) at any time point, indicating that the narrower SA lumen did not affect blood pressure in these mice.

### MC- and NK/MC-deficient mice produce growth-restricted pups

Because poor SA remodeling has been shown to be associated with fetal growth restriction^3^, we next asked whether NK/MC-deficient mice give birth to growth-restricted pups. Pups whose weight is below the 10^th^ percentile are defined as growth restricted, a special severity is present when pups weight is below the 5^th^ percentile^28^. We measured the placental and fetal weights and divided the latter by the former to calculate the feto-placental index (FPI) for all groups. We found a statistically significantly lower (*P* = 0.001) fetal weight at gd18 in NK/MC-deficient mice (1247 ± 113.3) compared to WT mice (1351 ± 115) (Fig. 3C). 4.8% of the fetuses from NK-depleted mothers had a weight below the 5^th^ percentile; 3.8% of the progeny from the MC-deficient mice were below the 5^th^ percentile and 7.5% were between the 5^th^ and 10^th^ percentiles. Of the fetuses from NK/MC-deficient mothers, 19.0% were below the 5^th^ percentile and 11.0% were between the 5^th^ and 10^th^ percentiles (Fig. 3F). Additionally, we found a statistically significantly higher (*P* = 0.05) placental weight at gd18 in NK-deficient mice compared to WT mice (Fig. S3). The FPI was statistically significantly decreased (*P* < 0.01) in the NK/MC-deficient mice (12.16 ± 2.843) compared with control (13.56 ± 1.498) (Fig. 3D). These results clearly indicate that both NKs and MCs participate in proper fetal development.

In the groups whose pups were naturally born, we found a significantly lower weight of pups (*P* = 0.05) in NK/MC-deficient mice (1340 ± 89.16) than in control mice (1402 ± 82.04) (Fig. 3E). The NK-depleted progeny exhibited a weight above the 10^th^ percentile, whereas 16.1% of the pups born to MC-deficient mothers were below the 5^th^ percentile and 9.7% were between the 5^th^ and 10^th^ percentiles. Notably, in mice lacking NKs and MCs, a much higher percentage (42.1%) of the pups were below the 5^th^ percentile (with 10.5% were between the 5^th^ and 10^th^ percentiles) than in the other groups (Fig. 3F).

NK depletion had a minimal impact on fetal weight; the absence of MCs had a negative effect on one of four pups at birth. The combined absence of NKs and MCs, however, impaired the growth of over half of the progeny. Taken together, our results show that both NKs and MCs are required for the attainment of normal fetal weight.

### Association between spiral artery remodeling and Mcpt5 expression by uMCs and uNKs

**Figure 4:**
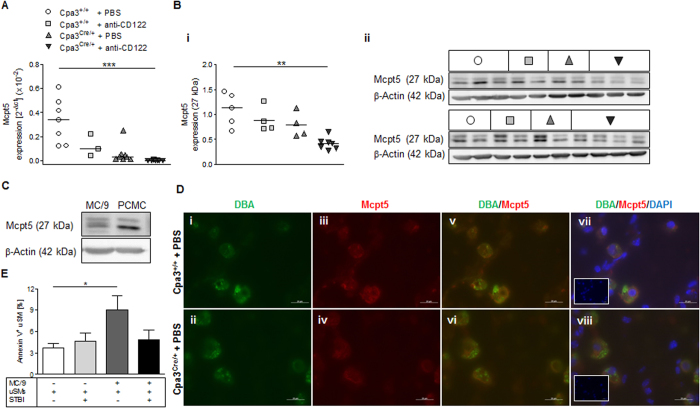
Mcpt5 expression in uMCs and uNKs as well as uSMC apoptosis induced by MC-derived chymase *in vitro*. **(A)** Real-time RT-PCR of decidual Mcpt5 mRNA at gd10. **(B)** Western blot analysis (i, relative expression; ii, blot) of decidual Mcpt5 protein (27 kDa) at gd10. β-actin (42 kDa) was used as a protein loading control. For (**A** and **B)**, the horizontal bars represent medians. Statistical differences were analyzed by using Kruskal Wallis followed by Dunns post test (**P < 0.01, ***P < 0.001). **(C)** Western blot analysis of Mcpt5 protein in MC/9 cells and PCMCs. **(D)** Immunofluorescence staining of uNKs (green, DBA lectin, i–ii), Mcpt5^+^ cells (red, DyLight™ 594, iii–iv) and nuclei (blue, DAPI, vii-viii) in implantation sections from Cpa3^+/+^ and Cpa3^Cre/+^ mice (scale bars = 20 μm). Overlay (v–viii) reveals Mcpt5 expression in DBA^+^ uNKs. Rectangles represent DAPI staining. **(E)** Flow cytometry analysis of the percentage of Annexin V^+^ cells following 48 h co-culture of uSMCs with MC/9 cells in the presence or absence of chymase inhibitor from *Glycine max* (Soybean) (STBI). Results from three independent experiments are presented as median + range. Statistical analysis was performed by using Kruskal Wallis followed by Dunns post test (*P < 0.05). gd, gestation day; PCMCs, peritoneal cavity mast cells; uSMCs, uterine smooth muscle cells.

Given that NKs and MCs are essential for SA remodeling and growth of the developing fetus, we asked which mediator could be involved in SA remodeling process. As we hypothesized that chymase could have an influence, differences in mouse α chymase Mcpt5 expression between the groups were analyzed. We found that mRNA and protein expression of the protease Mcpt5 (which is expressed by MCs)^29^ in the pregnant uterus (decidua) was diminished in the MC-deficient mice compared with controls. The lowest level of Mcpt5 expression was observed in mice lacking both uMCs and uNKs (Fig. 4A,B). We confirmed Mcpt5 expression in the mouse MC/9 cell line and in primary peritoneal cavity MCs (PCMCs) (Fig. 4C). Our finding that Mcpt5 mRNA and protein levels in the pregnant uterus were lower in the NK/MC-deficient mice than in MC-deficient mice (Fig. 4A,B) suggests that uNKs are a previously unknown Mcpt5 source.

To investigate whether the uNKs were also expressing Mcpt5, we double-stained implantations from MC-sufficient Cpa3^+/+^ and MC-deficient Cpa3^Cre/+^ mice with DBA FITC to specifically detect uNKs^30^ and Mcpt5. Interestingly, Mcpt5 and DBA co-localized in NKs at the feto-maternal interface in both MC-sufficient Cpa3^+/+^ and MC-deficient Cpa3^Cre/+^ mice (Fig. 4D). We found that uNKs were also positive for Mcpt5 in W-sh mice that are devoid of uMCs; these mice had an impaired SA shape^12^ (Fig. S4). Hence, Mcpt5 was expressed by both uMCs and uNKs, and its expression was very low in mice with suboptimal SA remodeling.

### MC chymase promotes apoptosis of uterine smooth muscle cells

We found a low Mcpt5 expression in mice with suboptimally remodeled SAs; we therefore hypothesized that the chymase Mcpt5 is involved in the remodeling of SAs. The SA remodeling process implies that uSMCs have undergone apoptosis^31^. It is known that uNKs are involved in this step through the secretion of chemokines and MMPs^32^. However, the role of uMCs and chymases in the uSMC apoptosis that occurs during SA remodeling has remained elusive. We therefore established an *in vitro* co-culture transwell system containing Mcpt5-expressing MC/9 cells and primary uSMCs in the presence or absence of soybean trypsin inhibitor (SBTI), which is used as a chymase inhibitor^33^,^34^,^35^,^36^,^37^. The addition of MCs to this transwell system provoked a statistically significant increase (*P* = 0.05) in the number of Annexin V^+^ uSMCs, and this increase was abolished by the addition of SBTI (Fig. 4E). These results confirm that an MC-derived chymase was responsible for uSMC apoptosis.

### Impaired spiral artery remodeling and fetal growth restriction in Mcpt5-deficient mice

**Figure 5:**
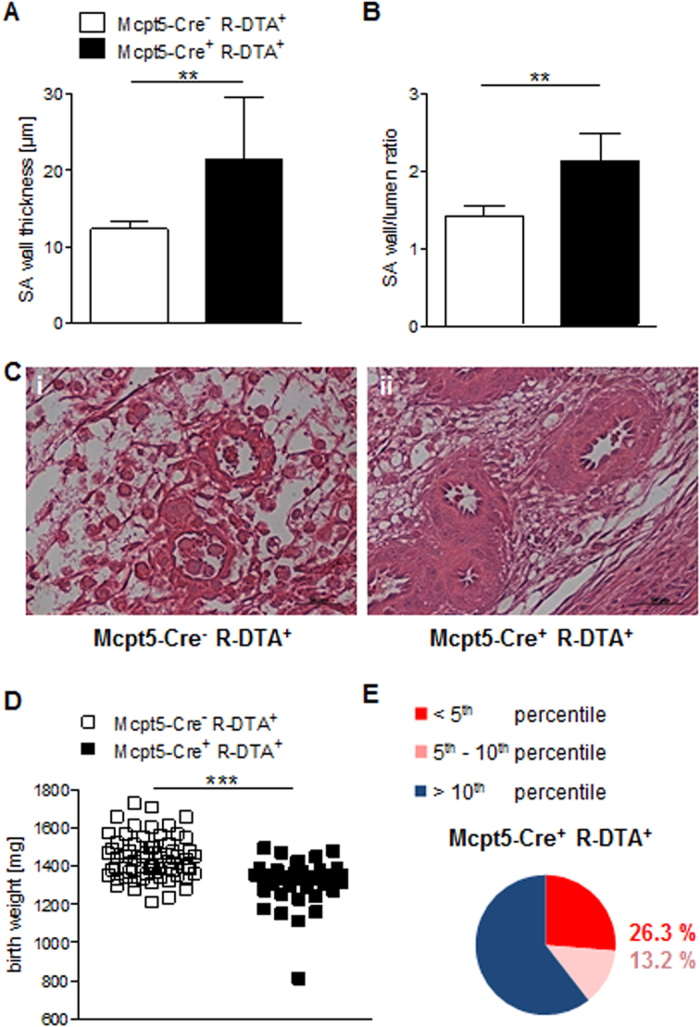
Impaired uterine spiral artery remodeling and fetal growth restriction in Mcpt5-deficient pregnant mice. SA wall and lumen diameters from 3 to 10 SAs in Mcpt5-Cre^−^ R-DTA^+^ control mice (*n* = 6) or Mcpt5-Cre^+^ R-DTA^+^ mice (*n* = 7) were measured. Based on the mean value obtained for each mouse, the means for the group were calculated and expressed as wall thickness **(A)** and the wall-to-lumen ratio **(B)**. Statistical differences were analyzed with the Mann-Whitney test (**P < 0.01). **(C)** Representative images of H/E-stained uterine sections from control (i) and Mcpt5-Cre^+^ R-DTA^+^ Mcpt5-deficient mice (ii, scale bars = 50 μm). **(D)** Birth weights of pups born to pregnant female controls (*n* = 66) or Mcpt5-Cre^+^ R-DTA^+^ mice (*n* = 38). Individual mice are depicted, with means. Statistical analysis was performed using the unpaired *t* test (***P < 0.001). **(E)** Pup weights from the Mcpt5-Cre^−^ R-DTA^+^ mice were classified as above the 10^th^ percentile (blue) or growth restricted (red, below the 5^th^ percentile; pink, between the 5^th^ and the 10^th^ percentile). Pups from wild type Mcpt5-Cre^+^ R-DTA^+^ females served as the basis for calculating the percentiles. SA, spiral artery.

Our data suggest that uterine Mcpt5-expressing innate immune cells are required for proper SA remodeling and unrestricted fetal development. However, by employing our *in vivo* model of NK/MC-deficiency, we cannot conclusively rule out the possibility that other molecules expressed in NKs and MCs are responsible for the observed phenotype. We therefore next investigated reproductive outcomes in Mcpt5-Cre^+^ R-DTA^+^ mice, which lack solely Mcpt5^+^ cells due to the cytotoxicity of Cre recombinase^38^. Pregnant Mcpt5-Cre^+^ R-DTA^+^ and Mcpt5-Cre^−^ R-DTA^+^ control mice showed comparable numbers of implantations (Fig. S5). SA remodeling was dramatically impaired in mice lacking Mcpt5^+^ cells, with a statistically significant increase in both SA wall thickness (Fig. 5A,C) and wall-to-lumen ratio (Fig. 5B,C) at gd10 (*P* < 0.01). These changes derived in a narrower lumen (Fig. 5C), and resulted in statistically significantly reduced (*P* = 0.001) fetal growth in Mcpt5-Cre^+^ R-DTA^+^ (1315 ± 19.74) compared to Mcpt5-Cre^−^ R-DTA^+^ control mice (1439 ± 13.66) (Fig. 5D,E). 26.3% of the Mcpt5-Cre^+^ R-DTA^+^ fetuses were below the 5^th^ percentile, and 13.2% were between the 5^th^ and 10^th^ percentiles. Thus, Mcpt5-expressing cells play a pivotal role in SA remodeling and fetal growth.

### Human uterine MCs secrete CMA1 and CMA1-containing MC supernatant as well as recombinant human CMA1 augment the migratory capacity of extravillous trophoblasts

**Figure 6:**
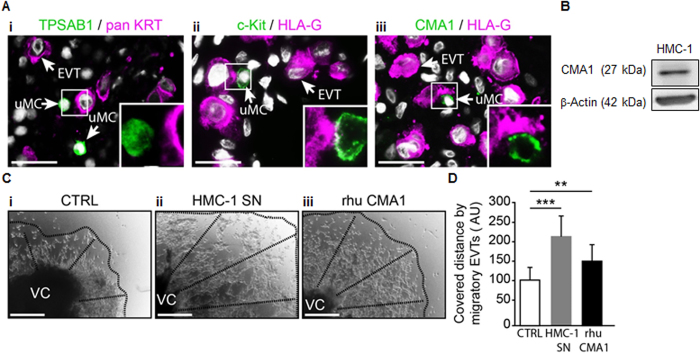
uMCs localize near invading trophoblasts in human first-trimester decidual tissue and MC-conditioned medium and human CMA1 increase the migration of extravillous trophoblasts *in vitro*. **(A)** Images depicting the close localization of uMCs and EVTs in first-trimester decidual tissue obtained from elective, legal pregnancy terminations. uMCs were stained for TPSAB1 (i), c-Kit (ii) or CMA1 (iii), and decidual EVTs were identified as pan KRT- (i) or HLA-G-positive (ii,iii) cells (scale bars = 50 μm). **(B)** Western blot analysis of CMA1 (27 kDa) in lysates from human HMC-1 MCs. β-actin (42 kDa) was used as a loading control. **(C)** Representative microscopic images of placental first-trimester villous explants cultured in the presence of control medium (i, CTRL), HMC-1-conditioned medium (ii, HMC-1 SN) or rhu CMA1 (iii) (scale bars = 100 μm). **(D)** The distance migrated by EVTs shown in **C** was calculated. Results are presented as means ± SEM. Statistical analysis was performed with two-way ANOVA followed by Sidak’s post-hoc correction (**P < 0.01, ***P < 0.001). EVTs, extravillous trophoblasts; rhu, recombinant human; u, uterine.

In mouse model, we found that chymase induces apoptosis of uSMCs, an important step in SA remodeling. Additionally, the specific deletion of chymase-expressing cells is associated with insufficient SA remodeling. Based on this, we hypothesized that chymase-expressing immune cells at the feto-maternal interface are required for successful SA remodeling. To understand whether these findings could have a broader relevance and be important for the human situation we first analyzed the localization of uMCs and their spatial relationship to EVTs in human first trimester decidual tissues by double immunofluorescence staining. This should provide insights whether chymase-expressing MCs would be theoretically able to influence EVTs *in vivo*. As suggested by previous *in vitro* observations^39^, maternal MCs that expressed mast cell tryptase (TPSAB1), c-Kit and CMA1 were located close to decidual EVTs (Fig. 6A). EVTs and MCs appeared to interact, as suggested by interception points between these cells (Fig. 6A). The close localization of uNKs and EVT has been documented already^40^. Some single CD56^+^ cells expressed c-Kit but there was no co-expression between TPSAB1 or CMA1 and CD56 (Fig. S6). Having seen that, we next asked whether chymase may also have a positive role in SA remodeling in humans. For this, we employed a well-established system^41^ to evaluate EVT migration that represents an important step before SA remodeling can take place^42^. We analyzed the effect of supernatant obtained from human HMC-1 cells (MC line expressing CMA1), that is the phylogenetic homolog of mouse chymase Mcpt5, (Fig. 6B) as well as the effect of human recombinant CMA1 on EVT migration. Also for this aspect in the human system, we focused on the role of MCs rather on NKs as the participation of NKs on human EVT outgrowth has already been widely documented^43^. To analyze the interplay between MCs and EVTs, we studied the influence of HMC-1-conditioned medium on human placental explants grown on Collagen-I. Strikingly, trophoblast outgrowth and migration were statistically significant increased (*p* < 0.001) in the presence of chymase-containing MC-derived medium (Fig. 6C,ii,D). We then analyzed the direct influence of the human chymase CMA1 (the) on trophoblast migration. We found that trophoblast outgrowth was statistically significant increased (*p* < 0.01) in response to CMA1 (Fig. 6C,iii,D). Thus, CMA1 is likely to play a pivotal role in human pregnancy by triggering the migratory potential of EVTs.

## Discussion

In this study we found that the combined ablation of uNKs and uMCs on the one hand and the selective deletion of Mcpt5^+^ cells on the other hand dramatically impaired SA remodeling, which had detrimental consequences for fetal growth. The importance of the additive effect of these two cell types is highlighted by the fact that their combined absence has a stronger effect on SA remodeling and fetal growth than the ablation of either cell type alone. Our data suggest a redundant role for both cells to ensure this important step in pregnancy. Their role is fulfilled through the secretion of the chymase Mcpt5. We found that MCs but also uNKs are a source of Mcpt5. Mcpt5 expression by uNKs was never reported before. The positive role of these cells on SA remodeling through Mcpt5 can be explained by the fact that Mcpt5 has a pro-apoptotic effect on uSMCs and apoptosis of these cells is required for SA. Our observations appear to be relevant for human pregnancies as the chymase CMA1- as well as CMA-1^+^ MCs are able to increase the migration ability of EVTs, a step that is relevant for the SA remodeling.

A successful pregnancy depends on the coordination of uterine and immune cells and hormonal changes that support tissue remodeling^44^. uNKs are important for SA remodeling; however, their absence does not impair pregnancy outcomes^7^. This finding supports the existence of redundant mechanisms that ensure the fulfillment of important processes, such as SA shaping, to guarantee blood supply from the mother to the fetus.

Fetuses from IL-15^−/−^ mice, which lack NK cells and some other cell types, only presented mildly compromised fetal weight compared with wild-type controls despite of having impaired SA remodeling^45^. The fact that NK-deficient mice do not have a strong phenotype of fetal growth retardation or maternal hypertension has puzzled scientists over the last few years^46^. Here, we report that NK-depleted Cpa3^+/+^ mice had a significantly increased placental weight in contrast to WT mice. Possibly, the higher placental weight compensates the decreased nutrient supply caused by the impaired SA remodeling. Increased placental weight in human is associated with a higher risk for behavioral disorders during childhood and cardiovascular disease in later life^47^. Increased placental weight may prevent fetuses from growth restriction as we observed that pups from NK-depleted mice were all above the 10^th^ percentile at birth. Our data suggests the existence of compensatory mechanisms in mice lacking NKs that prevent fetal growth restriction. A mediator involved in this mechanism may be secreted by the uMCs that are present in the NK-depleted mice. This notion is reinforced by the fact that the absence of both uMCs and uNKs resulted in growth restriction at birth that affected more than half of the progeny, whereas the lack of MCs alone caused one out of four of the pups to be growth restricted. In NK/MC-deficient mice, the degree of abnormal remodeling correlated with the severity of fetal growth restriction. Although more than half of the fetuses did not grow properly, their mothers did not develop gestational hypertension.

Our results show that NKs and MCs positively mediate SA remodeling through the expression of Mcpt5, a mouse α-chymase, which was almost absent in mice with disturbed SA remodeling. Chymases have several functions: they induce the accumulation of neutrophils, eosinophils and other inflammatory cells *in vivo*^48^, they degrade the extracellular matrix^22^,^23^ and they induce SMC apoptosis^22^,^25^,^26^. Chymases have been reported to be selectively expressed by MCs, where they accumulate in secretory granules^17^ and are secreted upon activation – although cellular sources for chymase secretion other than MCs have previously been suggested^49^,^50^,^51^. Here, we found a previously unreported source for Mcpt5: DBA^+^ uNKs. It is for sure interesting to investigate in future studies whether DBA^−^ uNKs, NKs or in general ILCs express Mcpt5 as well.

Chymase-secreting MC/9 cells stimulated the *in vitro* apoptosis of primary uSMCs, and the use of a specific chymase inhibitor abrogated this effect. The stimulation of apoptosis of primary uSMs by chymase-secreting cells, together with our observations of an insufficient SA remodeling in NK/MC-deficient mice and low uterine Mcpt5 levels, strongly indicate that chymases contribute to SA remodeling by initiating uSMC apoptosis. *In vivo*, MC-deficient Cpa3^Cre/+^ mice had more pronounced impairments in both SA remodeling and fetal growth compared with NK-depleted mice. On another note, levels of chymase expression in decidua were lower in MC-deficient than in NK-depleted mice. This finding may indicate that uMCs produce more Mcpt5 than uNKs do and may explain the more pronounced effect of MC absence. Alternatively, other chymases present in MCs may account for the positive effects of MCs on SA remodeling. When both cell types were absent and thereby their common chymase was missing, there was an exacerbation in the extent of growth restriction. To confirm the critical role of Mcpt5-expressing uMCs and uNKs for SA remodeling *in vivo*, we studied SA remodeling and pregnancy outcomes in mice lacking Mcpt5^+^ cells. These mice had severely impaired SA remodeling of a magnitude comparable to that of mice devoid of both uNKs and uMCs. Furthermore the weight of nearly 40% of the pups of Mcpt5-deficient mice was below the 10^th^ percentile. This result confirms the participation of Mcpt5^+^ cells in the remodeling of uterine SAs and, consequently in fetal growth. Interestingly, Mcpt5-Cre^+^ R-DTA^+^ mice presented less numbers of DBA^+^ uNKs when compared to the controls, even though we did not observe a complete absence of DBA^+^ uNKs (data not shown), which owes to the fact that uMCs are still the main producers of Mcpt5.

Mice data do not always mirror physiological relevant scenarios for the human situation. Here, we aimed to understand whether the findings reported here are of interest for human pregnancy. We especially concentrated on the participation of MCs and the chymases they secrete as the importance of uNKs is widely reported^8^,^42^,^52^,^53^. In samples from elective terminations of normally progressing first-trimester pregnancies, uMCs were located close to EVTs. We confirmed that human uMCs but not CD56^+^ NKs are producers of CMA1. Previous studies had already reported MCs at the feto-maternal^54^,^55^. However, without knowing their activation state or the mediators they secrete is not possible to predict its involvement in pregnancy success. We therefore studied the effect of human MC supernatant and recombinant chymase on the migration of invasive EVTs with a widely used system that assesses the ability of trophoblasts to migrate, thus indirectly addressing their ability to remodel SAs^41^,^56^. The addition of supernatant obtained from HMC-1 MCs, containing chymase, increased the migratory capacity of EVTs. This event is a necessary step for SA remodeling *in vivo*^57^. Recombinant human chymase CMA1 also provoked a significant increase in EVT migration. Hence, we confirm that human uMCs are located close to trophoblasts and contribute to their invasive features by secreting chymase. We hypothesize that chymase released by MCs has a positive influence on EVT migration and by doing so in SA remodeling.

Knowing the exact mechanisms and mediators that are responsible for a physiological SA remodeling is essential to help designing strategies that in the future could help preventing pregnancy pathologies associated with impaired SA remodeling. Based on this knowledge new therapeutic options can be developed for woman and their newborns suffering growth restriction.

## Materials and Methods

### Mouse models

C57BL/6J-Cpa3^Cre/+^ (Cpa3^Cre/+^) mice, which are deficient in connective tissue- (CTMCs) and mucosal-type-mast cells (MMCs), and their controls C57BL/6J-Cpa3^+/+^ (Cpa3^+/+^) were kindly provided by H-R Rodewald (Heidelberg, Germany)^27^. C57BL/6J-Mcpt5-Cre^+^ R-DTA^+^ (Mcpt5-Cre^+^ R-DTA^+^) mice, which lack Mcpt5-expressing cells, and their controls C57BL/6J-Mcpt5-Cre^−^ R-DTA^+^ (Mcpt5-Cre^−^ R-DTA^+^) were kindly provided by Prof. A. Roers (Dresden, Germany)^38^,^58^. Both strains were bred in our facilities. C57BL/6J-Kit^W-sh/W-sh^ (W-sh) mice were kindly provided by Dr. Marcus Maurer (Berlin, Germany). C57BL/6J mice were purchased from Janvier (Le Genest-Saint-Isle, France). Mice received food and water *ad libitum* and were maintained in our barrier facility with a 12-h light/dark cycle. Animal experiments were performed according to the institutional guidelines upon ministerial approval (Landesverwaltungsamt Sachsen Anhalt: 42502-2-1296UniMD). The experiments were conducted by authorized persons according to the Guide for Care and Use of Animals in Agriculture Research and Teaching.

Six- to eight-week-old females were allogeneically mated with BALB/c males (Charles River) and checked twice a day for a copulation plug. Plug appearance was defined as gestation day (gd) 0, after which the females were separated from the males. C57BL/6J females were sacrificed at gd10 to evaluate the depletion of natural killer cells (NKs). Cpa3 females were sacrificed at gd10 to verify the depletion efficiency of anti-CD122 and to harvest samples for analysis of implantation, uNKs and SAs and examination of Mcpt5 expression. After the blood pressure measurements, Cpa3 females from all groups were sacrificed at gd18 and the placental and fetal weights of the embryos were determined. A second set of Cpa3 females with all treatments was allowed to give birth naturally, which occurred between the end of gd18 and gd19, and the pup weights were determined. Mcpt5-Cre^+^ R-DTA^+^ and Mcpt5-Cre^−^ R-DTA females were euthanized at gd10 for implantation and SA analyses. A second set of females was allowed to give birth naturally and the pup weights were determined. W-sh and C57BL/6J controls were sacrificed at gd10 to examine Mcpt5 expression in the implantations.

### Collection of human tissue

First-trimester placental (*n* = 4) and decidual (*n* = 4) specimens were obtained from elective terminations of viable pregnancies between the 7th and 12th gestational weeks. The gestational age was determined by ultrasound. Patients were locally anesthetized and treated with misoprostol before surgical intervention. Tissues were collected with written informed consent, and the use of these samples was approved by the Ethics Committee of the Medical University of Vienna, Austria (084/2013). The study was conducted according to the Declaration of Helsinki. Tissues were employed for immunohistochemistry or *in vitro* studies.

### Cell lines

MC/9, a murine MC line, was purchased from ATTC and grown in RPMI 1640 (Gibco) supplemented with 10% FBS, 1% penicillin/streptomycin (P/S), 0.1 mM non-essential amino acids (Gibco), 0.05 mM 2-mercaptoethanol (Sigma) and 30 ng/mL IL-3 (Invitrogen). C57BL/6 primary uterine smooth muscle cells (SMCs) (Cell Biologics) were grown in a medium recommended by the company (Cell Biologics, cat. no. M2268-Kit).

HMC-1, a human MC line, was kindly provided by Dr. J.H. Butterfield (Rochester, USA) and was grown in IMDM (Gibco) supplemented with 1% P/S. The spent media supernatant was collected, diluted 1:3 with explant medium and used for *in vitro* experiments.

### Depletion of NK cells *in vivo*

Two different NK-depleting antibodies, anti-asialo GM1 (Wako) and anti-CD122 (BioLegend), were tested for their ability to deplete pNKs and/or uNKs *in vivo* in order to generate mice deficient in both MCs and NKs. The efficiency of pNK depletion was analyzed by flow cytometry. The presence or absence of uNKs was assessed by uNK-specific *Dolichos biflorus agglutinin* (DBA) lectin staining of implantations at gd10. For the anti-asialo GM1 treatment, C57BL/6J females were mated and anti-asialo GM1 (100 μL, 0.29 mg) was administered i.p. at gd0, 2, 4, 6 and 8. Control females received 100 μL of normal rabbit serum (Life Technologies) at the same time points. For the anti-CD122 treatment, anti-CD122 (250 μL, 0.25 mg) was administered i.p. to C57BL/6J females once at gd0, immediately after the detection of the copulation plug. Control females received PBS (250 μL), as previously described^59^,^60^. Mice were sacrificed at gd10 or gd18 and different organs were analyzed by flow cytometry to assess the pNKs; implantation sections from gd10 were analyzed by DBA lectin-specific staining to assess uNKs.

### Sample collection

Mice were anesthetized with a mixture of ketamine hydrochloride (Pfizer) and xylazine (Ceva), and blood was obtained by retro-orbital puncture. Mice were then sacrificed and the implantation rates were determined, as described previously^61^. For the flow cytometry analysis, the spleen and inguinal LNs were removed, washed with cold PBS and stored in cold RPMI until use. Two samples from the decidua were collected to isolate mRNA and proteins; decidua were washed in cold PBS, snap-frozen in liquid nitrogen and stored at −80 °C. For histological and immunohistochemistry studies, one implantation (gd10) per female was fixed in 4% PFA containing 0.1 M saccharose (pH 7.4) for 6 h at room temperature and embedded in paraffin after dehydration with ethanol and xylol cycles.

### Flow cytometry analyses and antibodies

Flow cytometry was performed to compare the numbers of pNKs in different organs from the mice in all groups. Single-cell suspensions were obtained from the spleen, blood, and inguinal LNs by crushing and filtering the tissue through a sterile net (100 μm) and lysing the erythrocytes with lysis buffer containing NH_4_CL, KHCO_3_ and EDTA. Cells were centrifuged, washed with RPMI and resuspended in FACS buffer. Single cell suspensions were obtained from the decidua by enzymatic digestion with RPMI supplemented with 5% P/S containing 0.005% liberase (90 min, 37 °C). After liberase inactivation with RPMI containing 10% P/S and 1% P/S, the tissue was crushed and filtered through a sterile net (100 μm), incubated with RPMI + 10% P/S + 1% P/S (30 min, 37 °C), centrifuged and the pellet was resuspended in FACS buffer. The cells were washed and incubated with antibody solution (1:100 with FACS buffer) for 30 min in the dark at 4 °C. The following antibodies were employed: FITC-conjugated rat anti-mouse CD122 (clone 5H4), PerCP-conjugated hamster anti-mouse CD3 (clone 145-2C11) and PE-conjugated mouse anti-mouse NK1.1 (clone PK136), all of which were purchased from BD Pharmingen. After staining, cells were washed and resuspended in 100 μL of FACS buffer and measured with a FACS Calibur (BD Biosciences). Data were analyzed with CellQuest Pro software 4.bf4b (BD Biosciences), and dot plots were made with GraphPad Prism v5.0.

### Quantification of uNKs with DBA lectin staining

Implantations were stained with DBA lectin to compare the numbers of uNKs between mice. After dewaxing and rehydration, slides were incubated with 3% H_2_O_2_ in methanol for 30 min at room temperature in a humid chamber to block endogenous peroxidases and then washed 2x with 50 mM PBS. After two steps of avidin and biotin blocking (15 min each), proteins were blocked by incubation with 1% BSA in 100 mM PBS for 30 min. Samples were stained overnight at 4 °C with DBA lectin (1:150 in 1% BSA/100 mM PBS). The next morning, samples were washed 2 × with PBS and treated with an HRP-solution for 30 min. After washing, samples were incubated with the AEC substrate chromogen (Dako) for 6 min and washed further. Counterstaining was performed with a hematoxylin solution for 1–2 min and a brief rinse in warm tap water. Slides were mounted with Aquatex^®^ and analyzed under a light microscope (Zeiss). uNKs were identified as DBA lectin-reactive, brown cells, and their numbers in each 1 mm^2^ area were calculated with the help of an eyepiece micrometer (Zeiss).

### Visualization of uMCs with toluidine blue staining

Mouse uterine sections were stained with toluidine blue to visualize the MCs. After standard dewaxing and rehydration procedures, slides were incubated in a 0.1% toluidine blue solution for 45 s and rinsed briefly in distilled water. Next, the slides were dehydrated by dipping them 10 times each into 75%, 95% and 100% ethanol and further incubating them in xylene 2x for 2 min each. Roti-Histokitt was used to cover the slides. uMCs were identified as blue cells with purple granules.

### Hematoxylin/Eosin staining

After standard dewaxing, slides were stained with hematoxylin for 2 min, rinsed 2x with warm tap water, stained with eosin for 1 min and finally rinsed in distilled water. After dehydration by dipping the slides 10 times each into 75%, 95% and 100% ethanol, the slides were incubated 2x in xylene for 2 min each. Slides were covered with Roti-Histokitt.

### Analysis of spiral arteries

SA features were measured in the H/E-stained samples. One to ten SAs per implantation per female were analyzed at gd10. The wall and lumen perimeters of the SAs were measured under a light microscope (200x magnification) with the AxioVision software v4 (Zeiss, Germany) according to the manufacturer’s recommendations. The SA wall and lumen diameters were calculated and expressed as wall-to-lumen ratios. Furthermore, the wall thicknesses were calculated. Then, the mean wall-to-lumen ratios or wall thicknesses of multiple SAs per female were calculated.

### Immunofluorescence

Immunofluorescence was performed on paraffin-embedded samples after standard dewaxing and rehydration. Antigens were retrieved in 0.1 M citrate buffer (pH 6.0) for 10 min in a microwave, and samples were then washed. For smooth muscle actin staining, slides were incubated overnight at 4 °C with an anti-smooth muscle actin antibody (Dako, clone 1 A4, 1:50 in 10% BSA in TBS), followed by washing and incubation for 2 h at room temperature with AF555-conjugated goat anti-mouse secondary antibody (Invitrogen, 1:500 in 10% BSA in TBS). For Mcpt5/DBA staining, slides were incubated overnight at 4 °C with an Mcpt5 antibody (Proteintech, 1:50 in 10% BSA in TBS), followed by washing and incubation for 2 h at room temperature with DyLight^™^ 594-conjugated donkey anti-rabbit secondary antibody (BioLegend, clone 4064, 1:500 in 10% BSA in TBS). Following washing, slides were incubated for 2 h at room temperature with DBA and horse gram FITC (US Biological, 1:50 in 10% BSA in TBS) and washed again. VECTASHIELD^®^ mounting medium with DAPI (Vector Laboratories) was used to counterstain the nuclei.

Human decidual tissues (10th–12th gestational weeks) were fixed with 7.5% formaldehyde and embedded in paraffin (Merck). Serial sections were dewaxed, and the antigens were retrieved by boiling in PT Module Buffer 1 (pH 6) (ThermoFisher Scientific) with a KOS MicrowaveStation (Milestone, Sorisole, Italy). Sections were blocked in 0.05% fish skin gelatin (FSG) (Sigma-Aldrich) and incubated overnight with the following antibodies: primary mouse anti-human antibodies directed against TPS1 (Dako, clone AA1, 1:100 in 0.05% FSG) or HLA-G (Exbio, clone MEM-G/9, 1:100 in 0.05% FSG) and rabbit anti-human antibodies against CMA1 (Sigma, 0.4 μg/mL in 0.05% FSG), c-Kit (R&D Systems, 5 μg/mL in 0.05% FSG) or pan KRT (GeneTex, 1:100 in 0.05% FSG). Appropriate isotype-specific control antibodies were used. Subsequently, the sections were incubated for 1 h at room temperature with goat anti-mouse or anti-rabbit IgG conjugated to Alexa Fluor^®^ 488 or 546 (Molecular Probes, 2 μg/mL in 0.05% FSG), counterstained with DAPI (Roche Diagnostics, 1 μg/mL in 0.05% FSG) and mounted in Fluoromount-G^®^ (SouthernBiotech, Birmingham, AL). Images were acquired on a BX50 fluorescence microscope equipped with a CC12 digital camera and Cell^P software (Olympus).

### RNA isolation, cDNA synthesis and quantitative real-time PCR

Total RNA was isolated using TRIzol^®^ reagent according to the manufacturer’s instructions. Briefly, frozen tissues were homogenized inTRIzol with an Ultra Turrax T25 homogenizer (NeoLab). RNA was extracted with chloroform and precipitated with isopropanol, washed with ethanol, and then diluted in RNA-free water. Finally, the RNA was quantified by measuring the UV absorbance at 260 nm, and a quality check was performed by measuring the absorbance at 280 nm, before storage at −80 °C.

For cDNA synthesis, 2 μg of total RNA was incubated with oligo dTs and RNAse-free water for 10 min at 75 °C. After 2 min on ice, the marked mRNA was incubated with dNTPs (2.5 mmol/L), DNase I (2 U/μL) and RNase inhibitor (40 U/μL) in reaction buffer for 30 min at 37 °C. DNAse was inactivated for 5 min at 75 °C and the samples were incubated on ice for 2 min. Reverse transcriptase (200 U/μL) and RNase inhibitor (40 U/μL) were added, and the cDNAs were synthesized at 42 °C for 60 min. After reverse transcriptase was inactivated for 5 min at 94 °C, the cDNA samples were stored at −20 °C.

Real-time polymerase chain reaction amplifications were performed with SYBR Green using an iQ5 Multicolor real-time PCR Detection System (Bio-Rad). Amplification reactions consisted of 1 μL of cDNA, 6.5 μL of SYBR Green PCR master mix (Applied Biosystems), 2 μL of RNAse-free water, 3 μL of Mcpt5 primer mix (forward primer, CCG ACA CAC TGC AGG AAG TA; reverse primer, TAT CCC AGC ACA CAG CAG AG) and 0.5 μL of fluorescein. The no template controls contained water instead of cDNAs. Reactions were performed in duplicate as follows: initial denaturation at 95 °C for 10 min, followed by 40 cycles of denaturation at 95 °C for 30 s, hybridization at 60 °C (for Mcpt5) for 45 s, elongation at 72 °C for 30 s and a final denaturation at 95 °C for 1 min. Samples were normalized to the housekeeping gene β-actin (forward primer, TGC GTC TGG ACC TGG CTG G; reverse primer, ATC CTG TCA GCA ATG CCT GGG).

### Protein isolation and western blotting

Tissues or cells were homogenized with an ultrasound homogenizer (BANDELIN electronic GmbH) in lysis buffer containing 10% NP40, 0.1 mg/mL n-Dodecyl-*β*-maltoside, 500 mM sodium fluoride, 10 mM sodium metavanadate, 100 mM PMSF, 1 M Tris (pH 7.5), 0.5 M EDTA and 5 M NaCl, followed by a 60 min incubation on ice to isolate the proteins. Afterwards, the cells were centrifuged and the supernatants were collected and stored at −80 °C overnight. Protein concentrations were determined by the Bradford assay according to the manufacturer’s instructions. Thirty micrograms of protein was separated on a 15% SDS gel and electrophoresed at 80 V. Proteins were transferred to a 0.45 μm nitrocellulose membrane in transfer buffer containing 20% methanol, 0.19 M glycine and 0.025 M Tris (pH 8.3) at 80 V for 2 h on ice. After blocking nonspecific-binding sites with 5% skim milk powder in TBS for 1 h, membranes were incubated overnight at 4 °C with primary antibodies against Mcpt5 (1:200 in 5% BSA in TBS/Tween), and then for 2 h at room temperature with a goat anti rabbit-HRP secondary antibody (Santa Cruz, 1:1,000 in 5% BSA in TBS/Tween).

### Blood pressure measurement

Maternal blood pressure was monitored throughout gestation to address whether insufficient SA remodeling provoked hypertension in mice. Non-pregnant females were conditioned to the blood pressure machine daily for 5 min each day for 2 weeks before starting the experiments to minimize their movements during the measurements. Blood pressure was non-invasively measured with a tail-cuff at gd 0, 2, 4, 6, 8, 10, 12, 14, 16 and 18 using TSE Blood Pressure Monitoring Systems (TSE Systems). Mice were warmed under an infrared lamp for 10 min before blood pressure was measured. Systolic and diastolic values were determined for each female by calculating the mean of 10 replicate values.

### Co-culture experiments

Mcpt5-expressing MC/9 cells (50,000) were co-cultured with uSMCs (50,000) to determine whether Mcpt5 influences the apoptosis of uSMCs. The experiment was performed in the presence or absence of trypsin inhibitor from *Glycine max* (soybean) (STBI, Sigma, 100 μg/mL), a known chymase inhibitor, to inactivate Mcpt5 in the system. A 6-well membrane insert system was used, which consisted of a 6-well plate and an insert with a pore size of 1 μm (Nunc), that prevented direct cell-to-cell contact between the MC/9 and uSMCs. After 48 h, transwells were removed and the uSMCs and supernatants were collected for annexin V/propidium iodide (PI) staining, which was performed according to the manufacturer’s instructions. Briefly, cells and supernatants were washed with cold PBS, resuspended in 50 μL of binding buffer and divided into two vials. One vial was stained with 1.25 μL of annexin V and 1.25 μL of PI; 2.5 μL of binding buffer was added to the second vial, which served as a negative control. The vials were incubated for 15 min in the dark. Afterwards, 100 μL of binding buffer was added, and the samples were measured with a FACS Calibur and analyzed to determine the number of annexin V^+^ cells.

### Weight determination

To determine weight differences among the fetuses or pups from all experimental groups, placental and fetal weights from fetuses at gd18 and from pups were measured by weighting each mouse with a micro scale (Kern & Sohn GmbH). When birth started (recorded by a baby phone camera, NUK, monitored at all times), we waited exactly 3 hours before measuring the birth weight, so that all pups were born and the mother was allowed to rest.

### Human first-trimester placental explants

Placental villi (7th–9th gestational weeks) were dissected and placed on Collagen-I drops (Corning), as previously described (Biadasie *et al*., 2014). Four replicate experiments were carried out with sixteen explants per condition. After 4 h, placental explants were covered with DMEM/Ham’s F-12 medium (Life Technologies) supplemented with 0.05 mg/mL gentamicin (Life Technologies). Subsequently, the placental explants were further incubated for 48 h in the presence of HMC-1-conditioned medium (1:3 in DMEM/Ham’s F-12 medium) or recombinant human CMA1 (Sigma, 300 ng/mL). Control explants were treated with vehicle or HMC-1-conditioned medium (1:3 in DMEM/Ham’s F-12 medium). The area of migrated EVTs was digitally photographed with a BX50 fluorescence microscope, and the EVT migration distance was quantified with Photoshop CS5 (Adobe). Three measurements per explant were collected with the ruler tool and mean values were calculated.

### Statistical analysis

Normality of the data sets was assessed with the D’Agostino Pearson-Omnibus test before analyzing the data with either parametric or non-parametric tests. Data are presented as medians or means (depending on their distribution) ± SEM. The number of mice or samples, the statistical test and the *P* values for the experiments are indicated in the figure legends. Statistical analyses were performed with GraphPad Prism 5.0.

## Additional Information

**How to cite this article**: Meyer, N. *et al*. Chymase-producing cells of the innate immune system are required for decidual vascular remodeling and fetal growth. *Sci. Rep.* **7**, 45106; doi: 10.1038/srep45106 (2017).

**Publisher's note:** Springer Nature remains neutral with regard to jurisdictional claims in published maps and institutional affiliations.

## Supplementary Material

Supplementary Figures
