# Supplementary material for: Chymase-producing cells of the innate immune system are required for decidual vascular remodeling and fetal growth
Source: Sci Rep. 2017 Mar 22;7:45106. doi: 10.1038/srep45106 (PMC5361184; doi:10.1038/srep45106)
Supplement: Supplementary Figures [file srep45106-s1.doc]

**SUPPLEMENTAL MATERIAL**

**Chymase-producing cells of the innate immune system are required for decidual vascular remodeling and fetal growth**

**Authors:** Nicole Meyer^1^, Katja Woidacki^1^, Martin Knöfler^2^, Gudrun Meinhardt^2^, Désirée Nowak^1^, Philipp Velicky^2^, Jürgen Pollheimer^2^, Ana C Zenclussen^1*^

**Affiliations:**

^1^ Experimental Obstetrics and Gynecology, Medical Faculty, Otto-von-Guericke University, Magdeburg, Germany

^2^ Dept. of Obstetrics and Fetal-Maternal Medicine, Medical University of Vienna, Vienna, Austria

*Corresponding author: Ana C. Zenclussen, Experimental Obstetrics and Gynecology, Medical Faculty, Otto-von-Guericke-University, Gerhart-Hauptmann Str. 35, 39108 Magdeburg, Germany. Phone: +49391/6717460, Fax: +49391/6717440, E-mail: [ana.zenclussen@med.ovgu.de](mailto:ana.zenclussen@med.ovgu.de)

Running title: Chymase influences vascular remodeling

**Supplementary Materials**

**
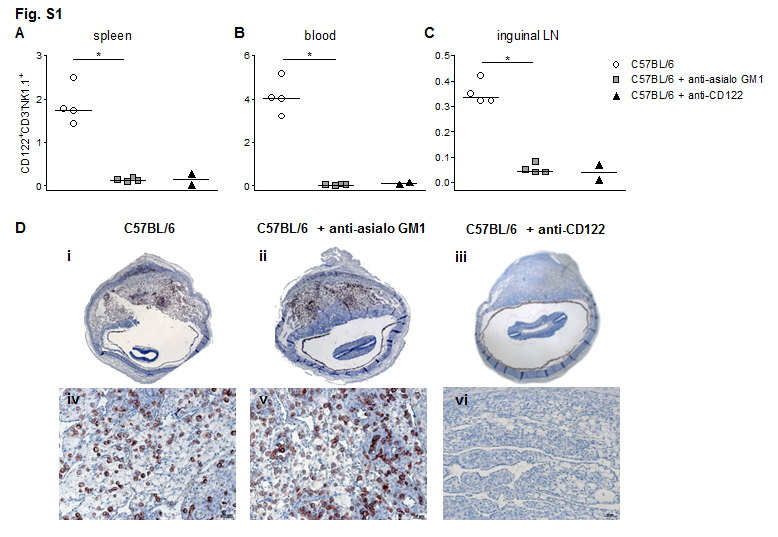
**

**Fig. S1: *In vivo* depletion of peripheral or uterine NKs with anti-asialo GM1 antibody and anti-CD122 antibody treatment.**

We treated C57BL/6J mice with anti-asialo GM1 (gd0, 2, 4, 6 and 8) or anti-CD122 (gd0) to test their effectiveness in depleting NKs**.** The percentages of CD122^+^CD3^-^NK1.1^+^ pNKs in the spleen **(A)**, blood **(B)** and inguinal LN (**C**) at gd10 are shown. Results are presented as individual values, and horizontal bars for the medians. Both antibodies depleted pNKs. Statistical analysis was performed using the Mann-Whitney U test (**P*<0.05). **(D)** uNKs were visualized by DBA lectin staining in implantation sections at gd10 to analyze the effectiveness of the antibodies at depleting the uNKs. The anti-asialo GM1 treatment failed to eliminate the NKs, and comparably stained fields can be observed in implantations of rabbit serum- (i, iv) or anti-asialo GM1-treated females (ii, v, 10x). Anti-CD122 treatment was effective at depleting uNKs, as no DBA^+^ cells could be observed in the anti-CD122-treated C57BL/6J females (iii, vi). Images in the lower panel (iv-vi) show a detailed view within the *decidua basalis* (scale bars=50 µm). uNKs are identified as DBA^+^, brown-stained cells. DBA, *Dolichos biflorus* agglutinin; gd, gestation day; LN, lymph nodes; p, peripheral; u, uterine.

***
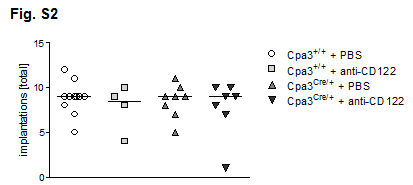
***

**Fig. S2: Total number of implantations in NK-depleted and MC- and NK/MC-deficient mice.**

A comparable number of implantations/female were found in PBS- or anti-CD122-treated Cpa3^+/+^ control mice and in PBS- or anti-CD122-treated MC-deficient Cpa3^Cre/+^ mice at gd10. Results are presented as medians with single values for each mouse and medians represented by horizontal bars. Statistical analysis was performed using the Kruskal Wallis followed by Dunns post test. gd, gestation day.


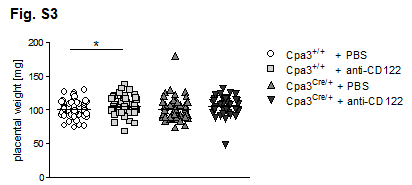


**Fig. S3: Placental weight in NK-depleted and MC- and NK/MC-deficient mice.**

Placental weights from the progeny of PBS- (*n*=64) or anti-CD122-treated (*n*=42) Cpa3^+/+^ females and PBS- (*n*=53) or anti-CD122-treated (*n*=42) Cpa3^Cre/+^ females at gd18. Results are presented as individual values for each placenta and means. Statistical differences were determined using One-way ANOVA followed by Bonferroni post-test (**P*<0.05).

***
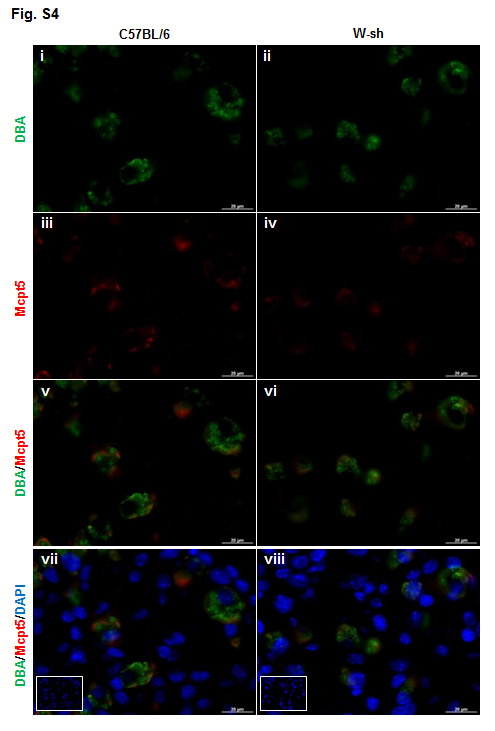
***

**Fig. S4: Co-expression of Mcpt5 and DBA lectin in uterine NKs from W-sh mice.**

Immunofluorescence staining was performed with DBA lectin (FITC, i-ii) to identify uNKs. Mcpt5 staining (DyLight™ 594, iii-iv) and DAPI counterstaining (blue, vii-viii) were performed in 5 µm paraffin-embedded sections of C57BL/6J and W-sh mice (scale bars=20 µm). W-sh mice contained Mcpt5^+^ cells, although they were devoid of uMCs. Co-staining with DBA lectin confirmed that uNKs were the source of Mcpt5 (v-viii). DBA, *Dolichos biflorus* agglutinin; u, uterine.


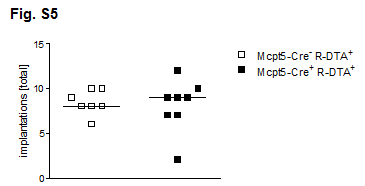


**Fig. S5: Total number of implantations in Mcpt5-deficient mice.**

Numbers of implantations in Mcpt5-Cre^-^ R-DTA^+^ and Mcpt5-Cre^+^ R-DTA^+^ mice at gd10 are depicted. Results are presented as single values with horizontal bars for the medians. Statistical analysis was performed with the Mann-Whitney U test. gd, gestation day.


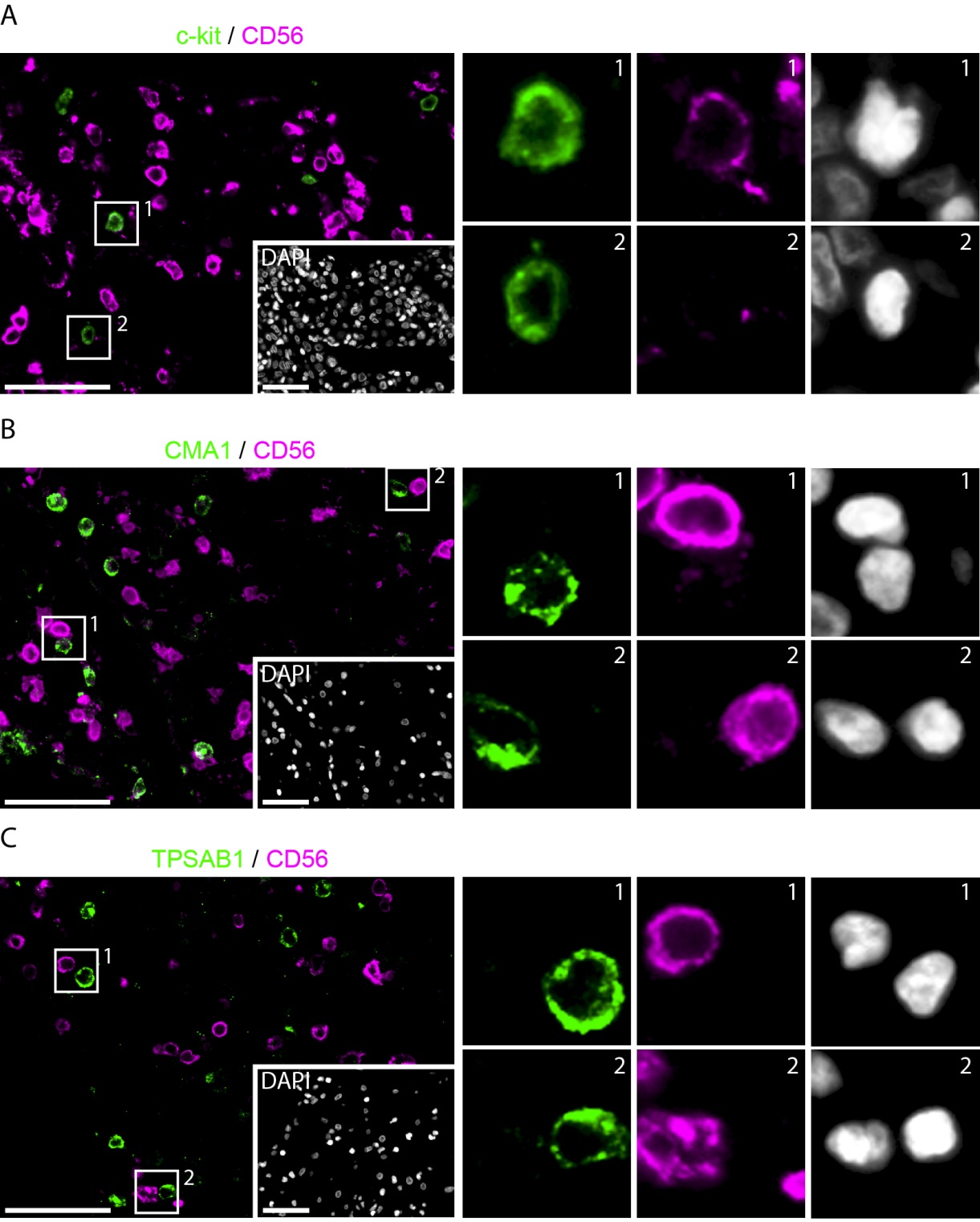


**Fig. S6 Few CD56^+^ uNK cells express c-kit, but all lack expression of CMA1 and TPSAB1.**

(A-C) Immunofluorescence co-stainings of CD56 and c-kit (A), CD56 and CMA1 (B), and CD56 and TPSAB1 (C) were performed on decidual tissue sections (9th week). DAPI nuclear staining is depicted in the lower right corner. Digitally zoomed insets display CD56^+^/c-kit^+^ (1) and CD56^-^/c-kit^+^ cells (2) (A), CD56^+^ or CMA1^+^ cells (B) and CD56^+^ or TPSAB1^+^ cells (C). Scale bars: 50 µm.
